# Traumatic Posterior Dislocation of Hip with Ipsilateral Fracture of Shaft of Femur: Temporary Fixator-assisted Reduction and Final Fixation with Interlocking Nail

**DOI:** 10.7759/cureus.5488

**Published:** 2019-08-26

**Authors:** Rajesh Rana, Saroj K Patra, Susanta Khuntia, Mantu Jain, Bishnu P Patro

**Affiliations:** 1 Orthopaedics, All India Institute of Medical Sciences, Bhubaneswar, IND; 2 Trauma & Orthopaedics, All India Institute of Medical Sciences, Bhubaneswar, IND

**Keywords:** fracture shaft of femur, ipsilateral hip dislocation, fixator, reduction

## Abstract

Ipsilateral fracture of the shaft of femur and dislocation of the hip are very rare injuries. There always exists a dilemma regarding the treatment to reduce hip and choosing the appropriate method of ﬁxation for a femur fracture, and a clear consensus is yet to be reached. A number of treatment methods such as the open reduction of femur and ﬁxation followed by hip reduction have been tried so far. Ipsilateral fractures and dislocation occur due to high-energy trauma, and reduction of hip dislocation is considered as an orthopedic emergency. Here, we report a case in which we tried a novel approach by temporarily ﬁxing the femur with an external ﬁxator and reducing the hip dislocation. In the next sitting, we performed femur ﬁxation in a closed manner with an interlocking intramedullary nail. We recommend that this novel method of treatment can be used for such types of injuries.

## Introduction

Ipsilateral fracture of the shaft of femur and dislocation of the hip are very rare injuries [1]. There always exists a dilemma regarding the treatment to reduce hip and choosing the appropriate method of ﬁxation for a femur fracture. These types of injuries occur due to high-impact forces which are common nowadays due to road traffic accidents. Here, we report the case of an 18-year-old male patient with ipsilateral hip dislocation and a fracture in the shaft of femur. The patient was treated with a temporary external ﬁxator followed by a reduction of hip dislocation. Finally, the ﬁxator was removed followed by a closed reduction and internal ﬁxation of the fractured shaft of femur with an interlocking nail [2].

## Case presentation

An 18-year-old male patient reported to our ED following a road traffic accident with severe pain in the right hip and right thigh. The patient had a deformity of the right hip and thigh, and was unable to walk. On clinical examination, we found that he had ﬂexion-adduction deformity of the right hip. His leg and foot showed external rotation and abduction. There was tenderness and abnormal mobility at the right thigh. In addition, there was tenderness at the right hip joint and the femoral head was palpable in the gluteal region. The range of movements at the right hip was grossly restricted and painful. There was no distal neurovascular deﬁcit noted.

**Figure 1:**
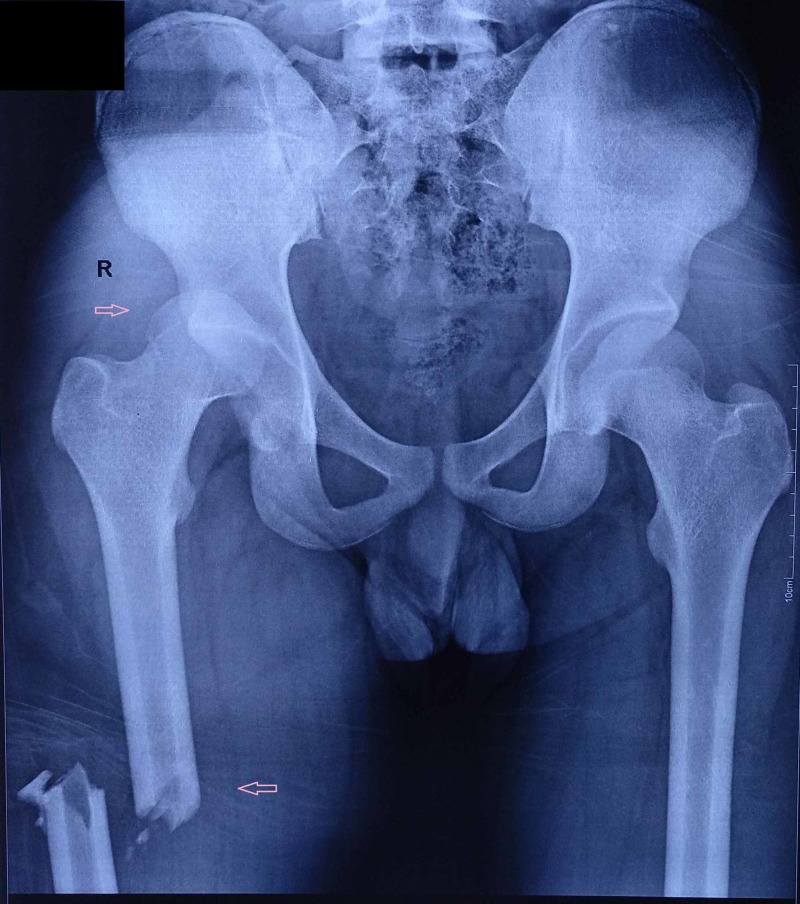
Preoperative radiograph.

After providing the advanced trauma life support in causality, the patient was sent for radiography. X-rays of the pelvis with both hips and of the right thigh with hip and knee were taken (Figure 1).

**Figure 2:**
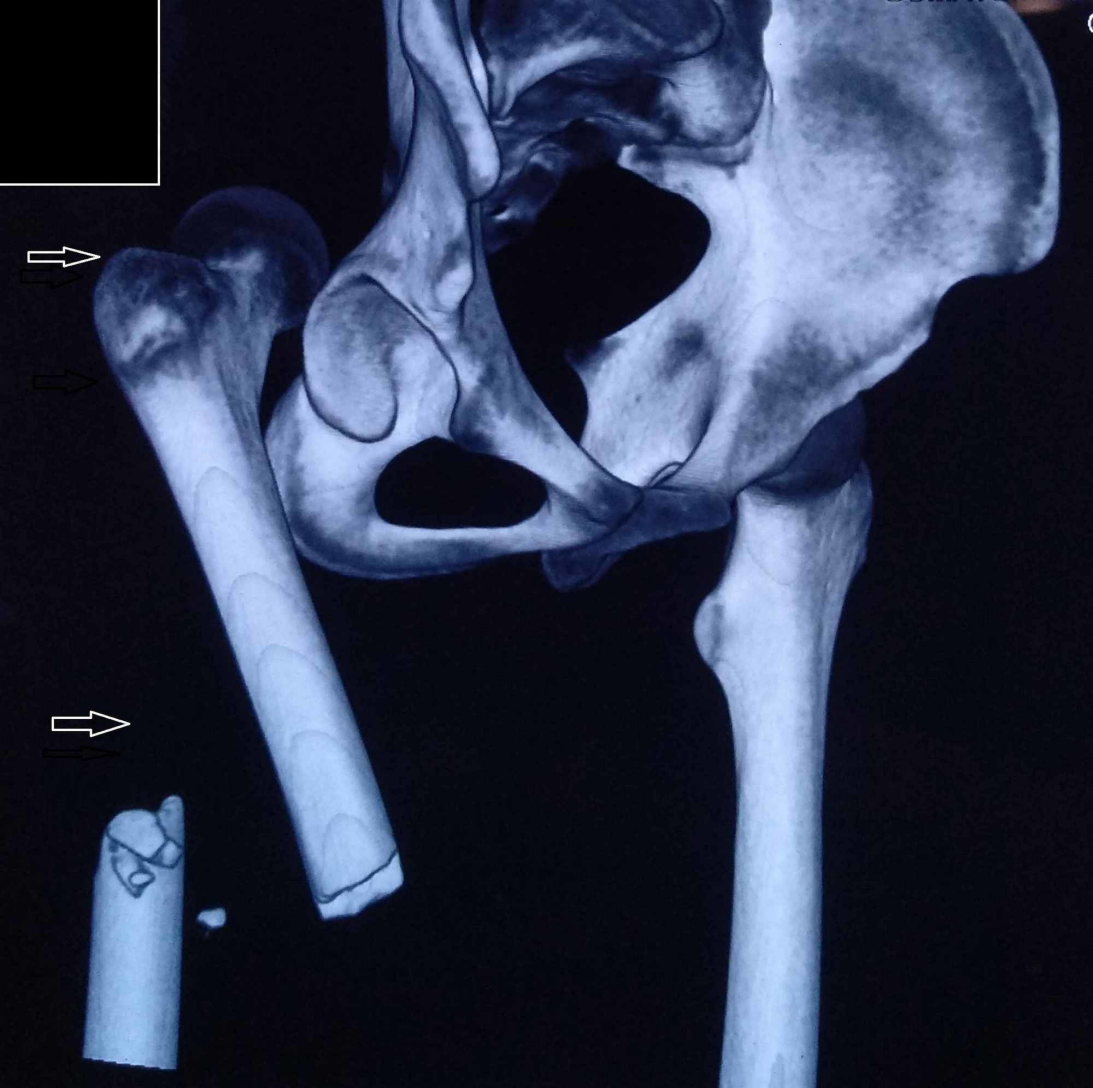
Preoperative CT scan.

CT scan of the hip and pelvis was also taken to exclude acetabulum fractures (Figure 2).

**Figure 3:**
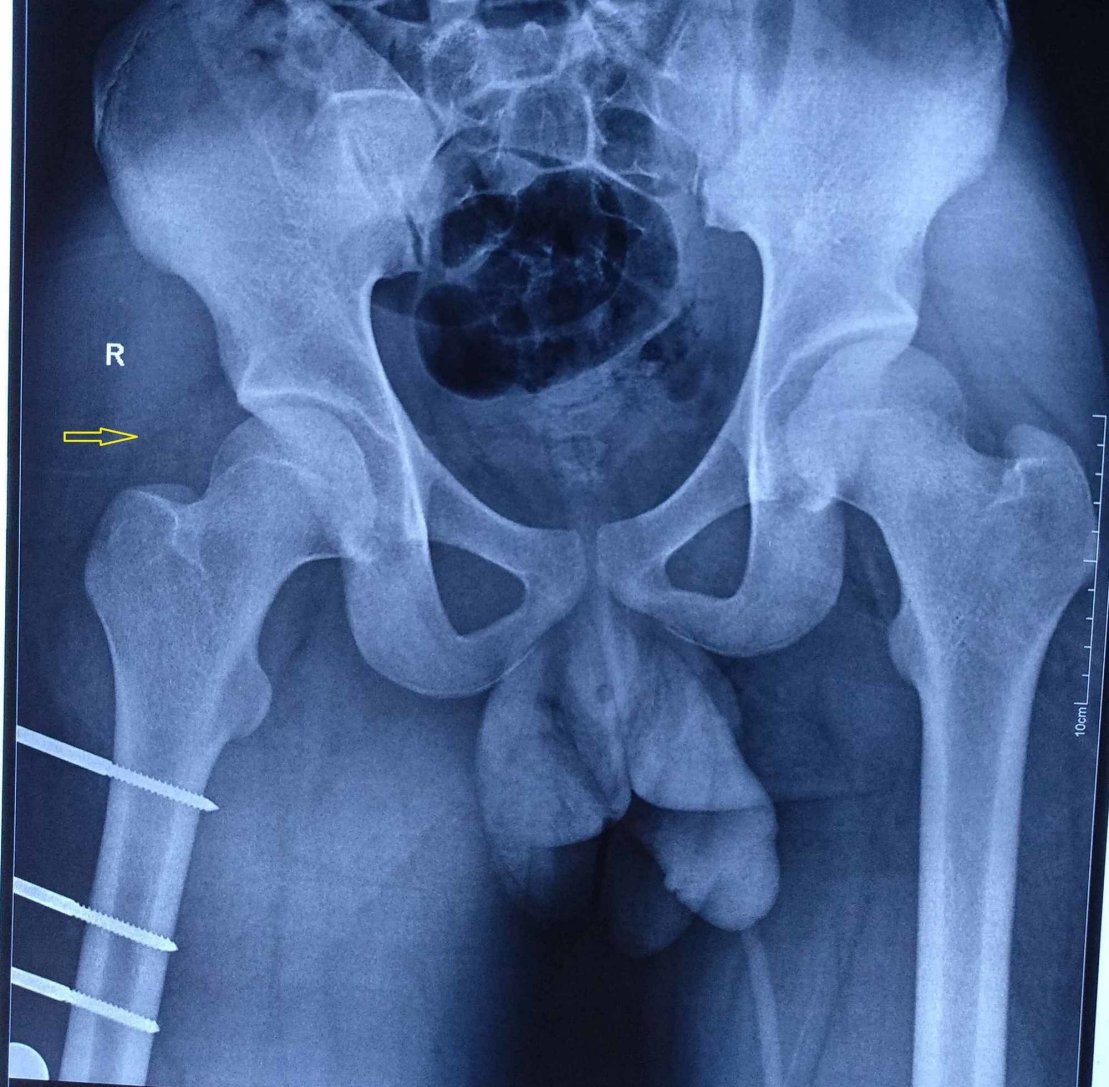
Reduced hip with external fixator in the femur.

From the radiograph, we conﬁrmed that there was a fracture in the shaft of femur and a posterior dislocation in the hip. The patient was taken to an emergency OT and subjected to closed manipulation of the hip joint. As there was a fracture in the shaft of femur, the force was not transmitted to the hip joint and we are unable to reduce the hip dislocation. At that moment, we had two options: open reduction and external ﬁxation, and closed manipulation of the dislocation of the hip joint. We placed a temporary external ﬁxator in the femur under anesthesia, and performed closed manipulation and reduction of dislocation of the hip using the Allis method (Figure 3).

**Figure 4:**
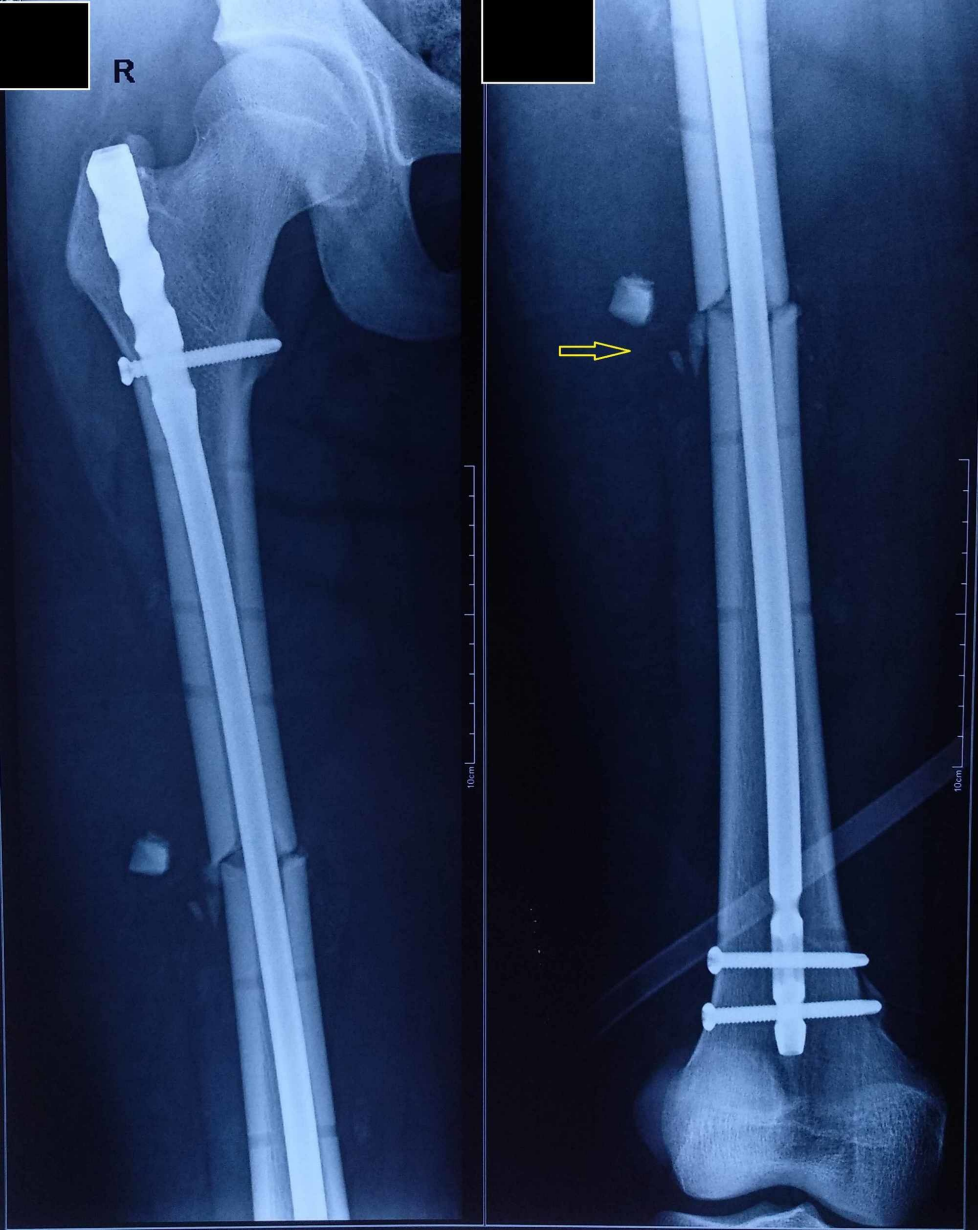
Postoperative radiograph with the intramedullary nail.

The patient was subjected to ﬁnal ﬁxation of the femur shaft fracture after one day in elective OT. Femur shaft fracture was ﬁxed with a closed reduction and an internal ﬁxation with an intramedullary femur interlocking nail (Figure 4).

The operation was done on a traction table under the guidance of ﬂuoroscopy.

Postoperatively, the patient’s legs were placed on an abduction pillow and physiotherapy was started. Low-molecular-weight heparin was given as deep vein thrombosis (DVT) prophylaxis. The patient was not allowed weight-bearing for two weeks. Gradually, he was allowed partial weight-bearing based on his tolerance.

## Discussion

Ipsilateral fracture of the shaft of femur and dislocation of the head are very rare injuries. So far, no consensus has been reached on the treatment of such injuries. Reduction of dislocation is considered an emergency; however, it is difficult [3] to perform because direct transmission of traction is not possible due to fracture in the shaft of the femur. This type of injuries generally occurs due to two types of forces: axial force that causes the hip dislocation and lateral force that causes fracture of the shaft of femur [4]. Dislocation of the hip is often missed to be diagnosed [5]. This can be avoided with a proper clinical examination and radiological investigation. Thus, CT scan of the pelvis and hip is always helpful in excluding other acetabulum or pelvic fractures.

Different methods such as placing Steinmann pin in the proximal femur and reduction have been tried by various authors. These are followed by open reduction and internal ﬁxation. Moreover, closed or indirect reduction is more favorable than open reduction unless there is a need for internal ﬁxation for an acetabular or proximal femur fracture. Many complications are often associated with an open reduction [6] and internal ﬁxation, which include hemorrhage, loss of fracture, infection, delay in union and avascular necrosis of the head of femur, and sciatic nerve injury [7]. In our case, using an external ﬁxator helped in indirect reduction of hip dislocation and precluded open reduction-related complications. The use of the fixator followed by closed reduction and internal ﬁxation with an interlocking nail gave satisfactory results without any complication.

## Conclusions

Ipsilateral fracture of the femur shaft and hip dislocation are rare injuries, and always difficult to be diagnosed and treated. These injuries are always considered as an orthopedic emergency. In such conditions, ﬁxator-assisted reduction of hip dislocation followed by closed reduction and internal ﬁxation with an intramedullary nail gives satisfactory results with minimal complications.
